# Multiorgan crosstalk in MASLD/MASH: from hepatic pathogenesis to systemic complications

**DOI:** 10.3389/fendo.2025.1720780

**Published:** 2025-12-18

**Authors:** Wenhua Bai, Zheng Zhu

**Affiliations:** Department of Diagnostic Radiology, National Cancer Center/National Clinical Research Center for Cancer/Cancer Hospital, Chinese Academy of Medical Sciences and Peking Union Medical College, Beijing, China

**Keywords:** MASLD, MASH, liver-brain axis, gut-liver axis, liver-kidney axis, multisystem complications, metabolic inflammation, therapy

## Abstract

Metabolic dysfunction-associated steatotic liver disease (MASLD) has evolved from a hepatic-centric condition to a systemic metabolic disorder, with multisystem complications driving clinical outcomes. This review comprehensively examines the pathogenesis and extrahepatic manifestations of MASLD, focusing on interorgan crosstalk. We first delineate the hepatic progression from steatosis to fibrotic metabolic dysfunction-associated steatohepatitis (MASH), emphasizing lipotoxicity, mitochondrial dysfunction, and inflammatory cascades. Subsequently, we analyze key extrahepatic axes (1): the liver-brain axis, where neuroinflammation and cognitive impairment are linked to hepatic metabolic disturbances (2); the gut-liver axis, highlighting roles of gut microbiota dysbiosis and intestinal permeability in disease progression; and (3) the liver-kidney axis, exploring shared fibrotic mechanisms and functional decline. Common pathways-including chronic inflammation, oxidative stress, and immune-metabolic dysregulation-underpin these systemic complications. Therapeutically, we advocate a shift from isolated liver-targeted approaches to integrated multisystem strategies. This review underscores the imperative to reconceptualize MASLD as a systemic disease, necessitating collaborative efforts to refine diagnostic frameworks and therapeutic paradigms for improving patient outcomes.

## 1 Introduction

Metabolic dysfunction-associated steatotic liver disease (MASLD), previously termed non-alcoholic fatty liver disease (NAFLD), represents the most prevalent chronic liver condition globally, affecting approximately one-quarter of adults and posing a substantial public health burden (1, 2). It is no longer viewed merely as a hepatic manifestation of metabolic syndrome but is increasingly recognized as a multisystem disorder characterized by complex interactions between the liver and extrahepatic organs (2). This paradigm shift underscores that the pathogenesis and progression of MASLD extend beyond the liver, involving intricate crosstalk with the gut, brain, and kidneys. The 2023 nomenclature update to MASLD further emphasizes its metabolic nature and provides a more accurate framework for understanding its systemic impacts (3).

The progression of MASLD to metabolic dysfunction-associated steatohepatitis (MASH) and fibrosis is driven not only by intrahepatic events but also by dysfunction in extrahepatic organs (4). The gut-liver axis plays a pivotal role, where dysbiosis and increased intestinal permeability allow translocation of pathogen-associated molecular patterns, promoting hepatic inflammation (5, 6). The brain-liver axis mediates neuroendocrine disturbances that exacerbate metabolic dysfunction and insulin resistance (7). Additionally, the kidneys are implicated through shared pathophysiological pathways, including oxidative stress and chronic inflammation, which accelerate both hepatic and renal damage (2, 8). These interorgan communications form a vicious cycle, propelling the disease toward irreversible stages.

Given the escalating burden of MASLD and its associated extrahepatic complications-including cardiovascular disease, chronic kidney disease, and cognitive impairment-there is an urgent need to elucidate the mechanisms by which extrahepatic organs contribute to MASH and fibrosis progression (6). Despite advances in pharmacological and lifestyle interventions, fully effective therapies remain elusive, partly due to an incomplete understanding of these systemic interactions. This review aims to synthesize current evidence on the roles of the gut, brain, and kidneys in MASLD pathophysiology, focusing on how each organ influences disease initiation, progression, and outcomes. Furthermore, we discuss emerging therapeutic strategies targeting these extrahepatic pathways and outline future research and clinical directions.

## 2 Hepatic pathogenesis: from steatosis to fibrotic MASH

The transition from simple steatosis to progressive MASH is orchestrated by a self-perpetuating cycle of metabolic insult, inflammatory amplification, and microenvironmental remodeling, ultimately driving irreversible hepatic fibrosis (1, 9, 10). This pathogenic cascade is initiated by a critical breakdown in hepatic lipid homeostasis, leading to the accumulation of excess triglycerides and cytotoxic free fatty acids within hepatocytes (>5% steatosis) (10). Lipotoxic stress overwhelms the capacity of mitochondria and the endoplasmic reticulum, culminating in profound oxidative stress and a surge of reactive oxygen species (ROS) (11). Beyond causing direct macromolecular damage, ROS serve as potent inflammatory second messengers, triggering the activation of resident Kupffer cells and the recruitment of peripheral immune cells (11, 12). These cells, in turn, unleash a torrent of pro-inflammatory cytokines (e.g., TNF-α, IL-6), which only exacerbate hepatocyte death but also set the stage for the next critical phase: fibrogenesis (13, 14).

A pivotal convergence point of this injury response is the activation of hepatic stellate cells (HSCs). In their quiescent state, HSCs store vitamin A; however, upon sustained exposure to ROS, inflammatory cytokines, and damage-associated molecular patterns (DAMPs) released from dying hepatocytes and activated macrophages, they undergo a dramatic phenotypic transformation into proliferative, fibrogenic myofibroblasts (15–17). Activated HSCs are the primary engines of fibrosis, depositing massive quantities of collagen and other extracellular matrix (ECM) components.

This process establishes a formidable vicious cycle: Activated HSCs themselves secrete pro-inflammatory and pro-fibrotic mediators, recruiting and activating more immune cells and perpetuating the inflammatory drive. Critically, the evolving fibrotic microenvironment itself becomes a major driver of progression. The accumulating ECM disrupts the normal architecture and function of Liver Sinusoidal Endothelial Cells (LSECs), leading to the loss of their characteristic fenestrations and capillarization (18, 19). This LSEC capillarization not only exacerbates hepatocellular injury by impairing nutrient and waste exchange but also directly perpetuates HSC activation (14), creating a feed-forward loop that renders the fibrotic process increasingly autonomous and irreversible.

The mode of hepatocyte death-whether apoptosis, necrosis, or necroptosis-further fuels this cycle (20). The release of cellular contents acts as a potent source of DAMPs, sustaining inflammation and providing continuous activation signals for HSCs. While the liver possesses inherent regenerative capacity, the relentless cross-talk between cell death, inflammation, and ECM deposition in MASH overwhelms these repair mechanisms, tipping the balance toward progressive scar formation.

**Figure 1:**
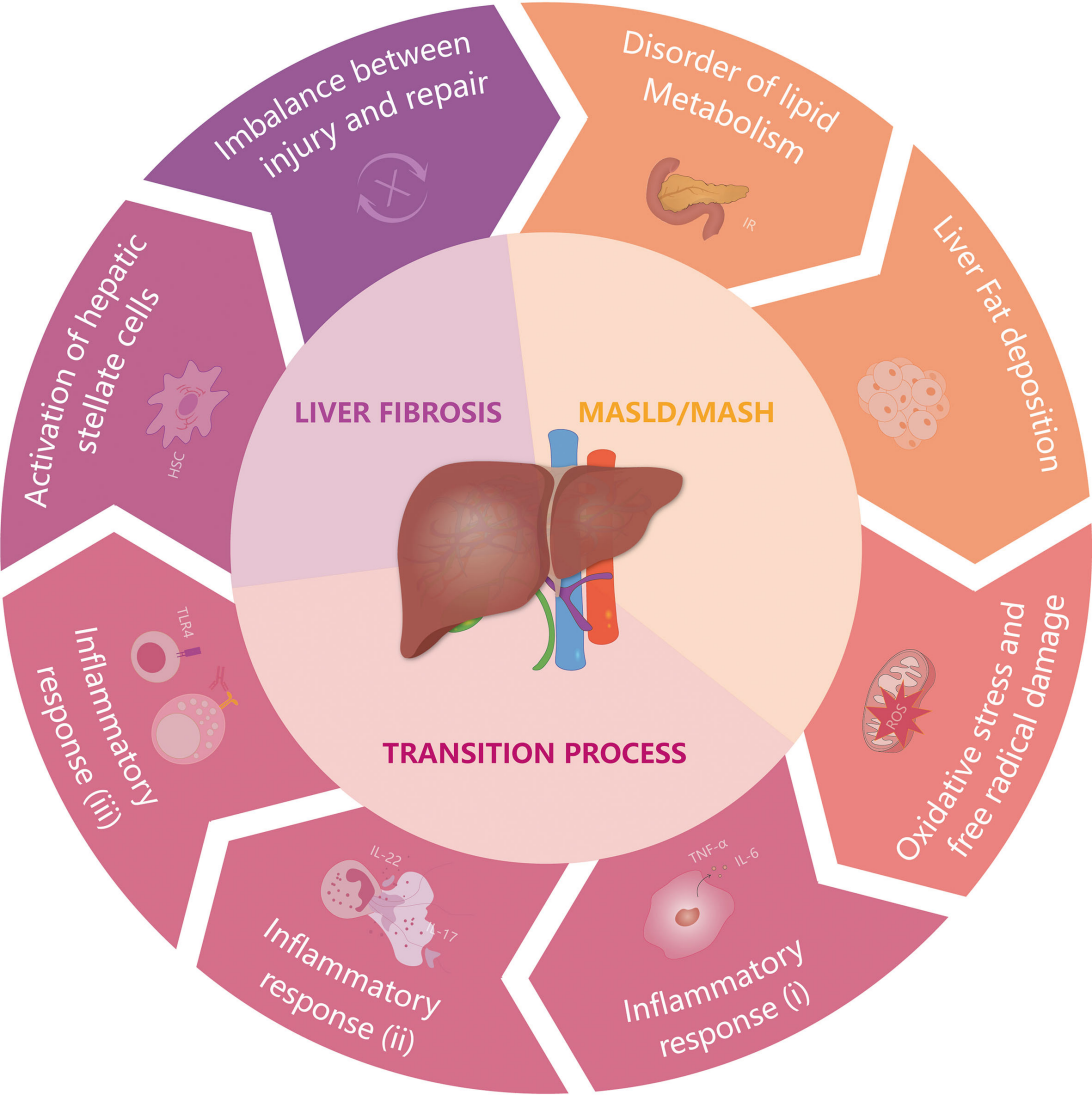
The occurrence and progression of MASH under the synergistic influence of multiple factors. The etiology and progression of non-alcoholic steatohepatitis (MASH) are multifaceted and intricate. Contributing factors include metabolic syndrome and type 2 diabetes, caused by insulin resistance (IR), which disrupt liver lipid metabolism and precipitate substantial hepatic fat accumulation. Subsequently, this condition engenders an overabundance of mitochondrial reactive oxygen species (ROS), resulting in oxidative stress that may impair mitochondrial function and lead to hepatocyte damage, culminating in cell death. The extensive demise of hepatocytes and ensuing liver damage provoke the mobilization and infiltration of immune cells, along with the activation of inflammatory pathways. For instance, the IL-22 and IL-17 cytokines, produced by neutrophils, stimulate neutrophil extracellular trap formation, which exacerbates liver injury and prompts an upsurge in pro-inflammatory cytokines, such as TGF-β and IL-6. This overexpression activates hepatic stellate cells (HSC), setting off a cascade that includes the production of the extracellular matrix, collagen deposition, and a disrupted equilibrium between tissue injury and repair. Progression to advanced liver fibrosis and cirrhosis constitutes the primary risk factors for hepatocellular carcinoma (HCC).

As fibrosis advances, the hepatic microenvironment undergoes profound transformations, including aberrant angiogenesis and altered hemodynamics (21). These changes mark the progression to cirrhosis, characterized by architectural distortion and functional failure. Furthermore, this pro-inflammatory, pro-proliferative microenvironment fosters a fertile soil for carcinogenesis, significantly elevating the risk of hepatocellular carcinoma (HCC) through mechanisms involving continued genomic damage and dysregulated cell signaling (22–24) (**Figure 1**).

## 3 MASLD as a multisystem disease: key inter-organ axes and their mechanisms

**Figure 2:**
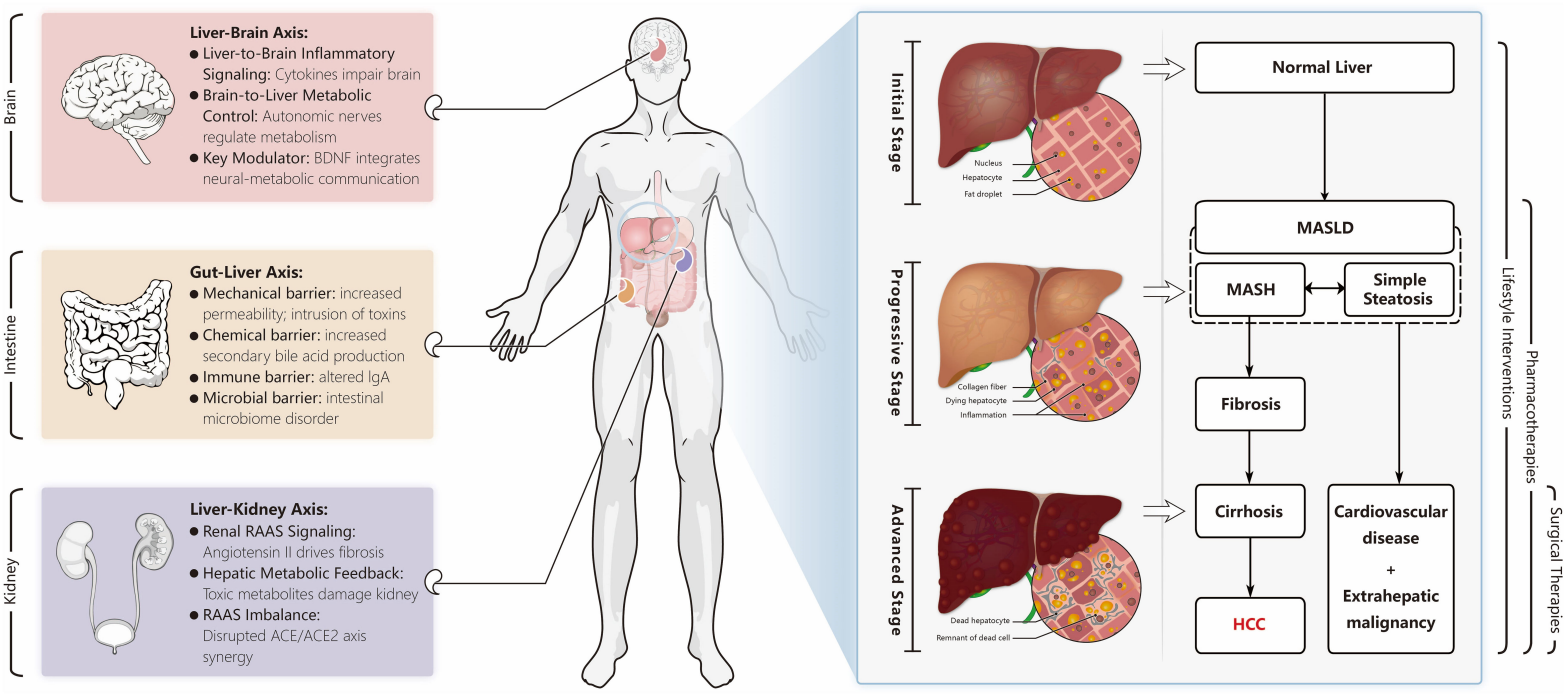
Natural history and treatment strategy of MASLD, mechanism of action of extrahepatic organs on MASLD evolution. Extrahepatic factors significantly impact the onset and progression of Non-Alcoholic Fatty Liver Disease (MASLD). Representative extrahepatic organs include the brain, intestine, and kidney. The brain maintains metabolic equilibrium via the neuroendocrine system, facilitated by Brain-Derived Neurotrophic Factor (BDNF). The intestinal barrier influences lipid metabolism and inflammation, while the kidney, through the Renin-Angiotensin-Aldosterone System (RAAS), plays a crucial role in liver fibrosis. These three organs work in concert to promote the development and advancement of MASLD. Under the influence of these extrahepatic and other factors, a healthy liver may transform into Non-Alcoholic Steatohepatitis (MASH) or simple steatosis, conditions collectively known as MASLD, distinguished by the presence or absence of intralobular inflammation. As the disease progresses, it can lead to liver fibrosis, cirrhosis, and potentially hepatocellular carcinoma (HCC). Simultaneously, exacerbation of MASLD may contribute to the development of cardiovascular disease and extrahepatic malignancies. Throughout the disease’s natural history, therapeutic interventions vary depending on the stage.

MASLD is recognized as a multisystem disorder that extends beyond hepatic manifestations to involve multiple organ systems, including the brain, intestine, and kidneys (**Figure 2**). This systemic involvement underscores the complex interplay between metabolic dysregulation, chronic inflammation, and neuroendocrine pathways in disease progression.

### 3.1 Liver-brain axis in MASLD

The interconnection between hepatic metabolic dysfunction and neurological impairment represents a critical aspect of MASLD pathophysiology. MASLD, regardless of severity, promotes systemic inflammation and metabolic anomalies that adversely affect cerebral function (25). Early-stage liver cirrhosis from MASLD has been confirmed to be associated with cognitive impairment (26, 27), behavioral alterations (28), and reduced total brain volume (29). In the late decompensated stages of the disease, hepatic encephalopathy is well-known to be a manifestation of its severity (9, 30).

This communication is fundamentally bidirectional. Efferent pathways from the brain regulate hepatic metabolism through autonomic nervous system outputs and neuroendocrine axes. The hypothalamus integrates peripheral signals to modulate energy homeostasis, influencing hepatic lipid metabolism and insulin sensitivity through sympathetic and parasympathetic outflow. Disruption of this brain-to-liver signaling, as evidenced in cancer cachexia studies where vagal dysfunction depletes hepatic HNF4α - a master regulator of metabolism - highlights the importance of intact brain-liver communication in maintaining metabolic homeostasis (31).Within this bidirectional network, brain-derived neurotrophic factor (BDNF) functions as a significant modulator at the interface of neural and metabolic processes. BDNF influences energy homeostasis through hypothalamic regulation of appetite and energy expenditure, enhancing leptin sensitivity while promoting sympathetic outflow that suppresses hepatic lipogenesis (32–35). Its role in glucose metabolism involves both potentiation of glucose-stimulated insulin secretion via pancreatic TrkB receptors and enhancement of peripheral insulin sensitivity, collectively ameliorating hepatic steatosis (36–38). Furthermore, BDNF supports vagus nerve-mediated anti-inflammatory pathways by maintaining afferent vagal fiber integrity essential for the inflammatory reflex (33), and modulating cholinergic signaling through α7nAChRs on hepatic macrophages, thereby constraining pro-inflammatory cytokine production (39, 40).

The role of neurotrophic factors in the liver is further supported by findings that BDNF and other neurokines (such as GFAP and GAP43) are significantly upregulated in fibrotic livers and contribute to HSC activation (41). Additionally, BDNF may influence liver fibrosis through its receptor p75, which is involved in regulating hepatic stellate cell differentiation and collagen production (42). Recent advances have identified additional molecular mediators in liver-brain communication. The integrated stress response in hepatic stellate cells - particularly through the noncanonical EIF3d-ATF4-S100P axis - drives metabolic reprogramming and liver fibrosis progression, establishing a pro-fibrotic hepatic environment that may adversely influence neurological outcomes (43, 44). On the therapeutic front, stimulating hepatic stellate cell-dependent extracellular matrix degradation via acid ceramidase inhibition and PKCα-ERK1/2-MMP-1 pathway activation offers a potential route for fibrosis resolution (45, 46).

The liver-brain axis operates through synergistic interactions where humoral, neural, and cellular pathways amplify each other’s effects. For instance, peripheral inflammation induced by hepatic damage can compromise blood-brain barrier integrity, allowing neurotoxic substances to enter the brain, while central nervous system responses further modulate hepatic inflammation and metabolism through autonomic outflow (47, 48). This reciprocal communication creates a self-reinforcing cycle that accelerates disease progression. Notably, liver-specific interventions such as siRNA targeting Cyclin M4 have been shown to reverse MASLD-associated social memory and sensorimotor deficits, restoring hippocampal synaptogenesis and mitochondrial function, which underscores the causal and therapeutically targetable nature of the liver-brain axis in MASLD-related neurological complications (49).

A deeper understanding of these liver-brain interactions promises to reveal novel therapeutic strategies for MASLD by simultaneously addressing both hepatic and neurological aspects of the disease.

### 3.2 The gut-liver axis: role of microbiota and intestinal barrier

In healthy individuals, intricate communication exists between the liver and the gut, enabling the formation of functional units known as enterohepatic axes. Anatomically, portal circulation links the liver and the gut, while the intestinal barrier restricts their direct connection. Nevertheless, in certain instances, excessive fat buildup resulting from alcohol or drug misuse, a high-fat diet, and intestinal inflammation can lead to alterations in intestinal epithelial cells and mucosa. These changes lead to the destruction of the intestinal barrier, the change of intracellular connectin, the increase of intestinal permeability and the disturbance of intestinal microbiota through various factors, thereby facilitating the onset and progression of MASLD (5, 50).

We next examine the breakdown of the intestinal mucosa’s mechanical barrier. This breakdown can be attributed to factors such as a high-fat diet, which can lead to intestinal microbial disorders, fostering pathogen growth and impairing the mucosal barrier. Inflammatory conditions also contribute to the contraction and relocation of tight junction (TJ) proteins, enlarging the TJ pores and increasing permeability (51). Consequently, gut bacteria and their byproducts, including endotoxins, are translocated through the portal system into the blood and liver, activating Kupfer cells and exacerbating the disease (50). Recent investigations into therapeutic strategies have demonstrated that compounds like ACT001 can alleviate MASLD by specifically restoring intestinal barrier integrity, highlighting the potential of targeting the mechanical barrier for intervention (52).

The significance of the chemical barrier within the intestinal tract cannot be overlooked, as it serves to protect the mucosal lining from the assault of microorganisms and enzymes. This barrier comprises substances such as gastric acid, mucus, mucin, bile, bile acids, glycosaminoglycans, digestive enzymes, lysozyme, and antimicrobial peptides. Among these, bile acids have garnered considerable attention due to their significant association with the development and progression of MASLD (53). A diet high in fat can alter the composition of the gut microbiota, leading to an increase in secondary bile acids, particularly deoxycholic acid, which contributes to microbial imbalance in the gut and promotes the progression of MASLD (54, 55). The molecular basis for this link is further elucidated by findings that certain interventions, such as ACT001, facilitate the generation of uncombined bile acids and their accumulation in the ileum, thereby downregulating the enteral Farnesoid X Receptor (FXR)-FGF15 pathway and contributing to MASLD improvement (52).

Next, the disruption of the intestinal immune barrier occurs. The key component of this barrier is IgA, which is secreted by lymphocytes and plasma cells. IgA specifically targets Gram-negative bacteria found in the gastrointestinal tract. However, when the intestinal mucosa is damaged, the effectiveness of IgA is compromised, leading to the facilitation of bacterial translocation within the intestine and contributing to inflammation development (50, 53, 56). Beyond impaired humoral immunity, cellular immune pathways also play a critical role. It has been shown that gut-primed neutrophils, assisted by intraepithelial lymphocytes, can migrate via the portal vein and release neutrophil extracellular traps in the liver, which subsequently activate Kupffer cells and exacerbate hepatic inflammation and injury, establishing a direct cellular link between intestinal and liver inflammation (57). Furthermore, intestinal inflammation raises intestinal permeability, facilitating the passage of bacteria and bacterial products like LPS into the bloodstream, thereby triggering systemic inflammatory responses, including in the liver (5, 58, 59).

It has to be mentioned that the gut microbial barrier is broken. A high-fat diet causes a disorder in the gut microbiome, altering its composition, increasing the proportion of pathogenic and gram-negative bacteria and decreasing the proportion of beneficial bacteria (53). The imbalance of intestinal microbes leads to changes in the intestinal microenvironment, such as reducing intestinal pH, increasing intestinal oxidative stress, and further damaging the intestinal barrier (60, 61). Owing to the variegated composition of the gut microbiota, it holds promise as an emergent biomarker and therapeutic target for MASLD. Interventions targeting the gut microbiota, encompassing antibiotic/probiotic therapies and fecal microbiota transplantation, have burgeoned as innovative strategies for the prevention and management of MASLD (62–64). Evidence from recent studies has revealed the role of specific microbial metabolites in this process. A notable example is the microbe-derived bile acid 3-succinylcholic acid (3-sucCA), which has been shown to hinder the progression of MASLD (65). Its protective effect is not mediated through conventional bile acid receptors like TGR5 or FXR, but rather through a positive modulation of the gut microbial ecosystem. Specifically, as a gut-restricted metabolite, 3-sucCA fosters the proliferation of the beneficial bacterium *Akkermansia muciniphila*, thereby reinforcing the integrity of the gut barrier and illustrating a novel mechanism of microbe-host interaction (65, 66).

Intestinal barrier damage and liver damage caused by different mechanisms interact. Damage to the intestinal barrier causes enterogenic inflammation and bacterial products to enter the liver and activate hepatic astrocytes, further promoting the occurrence of liver fibrosis and inflammation. Conversely, liver-derived inflammatory mediators and altered bile acid profiles can feedback to the gut, further compromising intestinal barrier integrity and amplifying the local inflammatory response. The occurrence and progression of liver disease further damage the intestinal barrier function, forming a vicious cycle.

### 3.3 The liver-kidney axis: shared mechanisms of fibrosis and dysfunction

The reclassification from NAFLD to MASLD necessitates a reassessment of the epidemiological patterns and interrelations with other chronic conditions (67, 68). In this context, the intimate link between MASLD and Chronic Kidney Disease (CKD) has emerged as a significant area of focus. This connection can be attributed to a constellation of overlapping risk factors, notably Type 2 Diabetes Mellitus, obesity, dyslipidemia, and insulin resistance. Evidence from large-scale epidemiological analyses indicates that although the association between MASLD and CKD is partly mediated by insulin resistance, the severity of liver fibrosis remains an independent risk factor for renal impairment, underscoring the complex interplay between shared metabolic derangements and direct organ injury (69). Furthermore, both diseases exhibit convergent molecular mechanisms, characterized by inflammation, oxidative stress, and fibrosis, which are ubiquitous features in MASLD and CKD, thereby reinforcing their interconnection (70–72). Remarkably, recent findings suggest a genetic predisposition to MASLD, potentially mediated by specific gene polymorphisms, such as the PNPLA3 rs738409 G allele, which might predispose individuals to renal dysfunction (67). This insight implies a potential bidirectional relationship between MASLD and CKD, indicating that MASLD’s progression could influence CKD onset and vice versa (71).

Kidney factors impact the development and progression of MASLD via the renin-angiotensin-aldosterone system (RAAS). Conversely, liver-derived factors can promote CKD through diverse pathways, suggesting a bidirectional pathogenic relationship (67, 73–76). Liver cells, such as hepatocytes, hepatic stellate cells, and Kupffer cells, express receptors for angiotensin and mineralocorticoid, components of the RAAS that are implicated in fibrotic inflammation within MASH (77–79).

Mineralocorticoid receptor (MR) activation is a determinant in the pathophysiology of various diseases, including MASLD (67, 80, 81). In studies have demonstrated that aldosterone can increase insulin resistance (IR) in adipocytes and hepatocytes by degrading IRS-1 and IRS-2, as well as by impairing normal adipocyte differentiation and function (79, 82). Additionally, aldosterone exerts multifaceted effects on immune cells expressing MR. Activation of MR in immune cells stimulates an inflammatory response, with MR activation in macrophages inducing a shift towards an inflammatory phenotype. In CD4+ lymphocytes, MR activation promotes differentiation into pro-inflammatory Th17 cells, thereby influencing the function of dendritic cells. Similarly, MR activation induces cytotoxic CD8+ T lymphocytes. These responses establish a pro-inflammatory environment in the liver, leading to increased secretion of IFN-γ and affecting hepatocyte lipid accumulation (83). In contrast, experimental studies have shown that MR antagonism provides a hepatoprotective effect against steatosis and fibrosis in NASH, which underscores the role of MR in promoting the development and progression of MASLD (84, 85).

Alternatively, research indicates that angiotensin II is a pivotal player in the progression of liver inflammation and fibrosis, with altered TLR4 and sphingosine kinase 1 (SphK1)/sphingosine 1-phosphate (S1P) signaling pathways being key contributors to the pathogenesis of MASLD (77). This suggests that angiotensin-converting enzyme inhibitors (ACE-Is) and angiotensin receptor blockers (ARBs) may represent effective therapeutic options for patients with MASLD (67, 86). ARBs mitigate insulin resistance by inhibiting the RAAS, leading to decreased cytokine production (including tumor necrosis factor-α), elevated adiponectin levels, and enhanced pancreatic insulin secretion and cellular insulin signaling. ARBs also exert anti-fibrotic effects by inhibiting the activation of hepatic stellate cells and reducing the secretion of pro-fibrotic cytokines such as transforming growth factor-β (87). Emerging therapeutic strategies are exploring the potential of activating the protective ACE2-Ang-(1–7)-Mas axis to restore RAAS balance and mitigate disease progression in both the liver and kidneys. The role of bioactive metabolites and the gut-liver-kidney axis is also gaining recognition (88). Accumulating evidence highlights that alterations in microbiota-derived metabolites, including bile acids, provide a crucial link between MASLD and CKD, suggesting that interventions targeting the gut microbiota may offer benefits for both organs (89).

Activation of the RAAS promotes oxidative stress, inflammation, and fibrosis, making the study of RAAS antagonists a potential treatment option for MASLD. The intricate crosstalk within the kidney-liver axis, mediated by an imbalanced RAAS and other factors, underscores the need for a holistic approach to managing patients with MASLD, particularly those with concurrent CKD.

### 3.4 Common mechanisms of systemic complications: beyond individual organ axes

While the liver-brain, gut-liver, and liver-kidney axes illustrate organ-specific pathways, the systemic progression of MASLD/MASH is driven by a convergence of overarching pathological mechanisms that transcend individual organs. These shared mechanisms-chronic inflammation, metabolic dysregulation, oxidative stress, and immune activation-form a synergistic network that amplifies disease severity across multiple systems (2, 90). Understanding these common pathways is crucial for developing holistic therapeutic strategies that target the disease’s multisystemic nature. These shared mechanisms and their systemic manifestations are summarized in **Table 1**.

**Table 1:** Expression patterns of genes associated with disease occurrence and progression via different pathways.

Mechanism	Liver	Brain	Gut	Kidney
Chronic Inflammation	Hepatocyte injury, DAMP release, Kupffer cell activation	Blood-brain barrier dysfunction, microglial activation, neuroinflammation	Intestinal barrier disruption, dysbiosis, endotoxemia (LPS)	Renal endothelial injury, tubulointerstitial fibrosis
Oxidative Stress	Mitochondrial dysfunction, ROS overproduction, lipid peroxidation	Neuronal oxidative damage, compromised synaptic function	Intestinal epithelial apoptosis, increased permeability	Glomerular sclerosis, tubular injury
Immune Dysregulation	Innate immune activation (Kupffer cells), adaptive responses (Th17)	Microglial priming by liver-derived cytokines, altered BDNF signaling	TLR4 priming by gut-derived endotoxins, compromised IgA defense	TLR4 signaling activation, inflammatory cell infiltration
Metabolic Dysregulation	*De novo* lipogenesis, Insulin Resistance (IR)	Hypothalamic appetite dysregulation, bile acid (FXR) signaling	Impaired barrier function, microbial metabolite actions (e.g., 3-sucCA)	Sodium retention, hypertension, altered RAAS (e.g., ACE2-Ang-(1-7)-Mas axis)

Chronic low-grade inflammation serves as a cornerstone of systemic complications. Hepatocyte injury and lipotoxicity in MASLD trigger the release of pro-inflammatory cytokines (e.g., TNF-α, IL-6, IL-1β) and damage-associated molecular patterns (DAMPs), which enter the systemic circulation via compromised hepatic sinusoids (91). This inflammatory milieu not only perpetuates hepatic steatosis and fibrosis but also contributes to blood-brain barrier dysfunction, neuroinflammation, and cognitive decline; intestinal barrier disruption and dysbiosis; and renal endothelial injury and tubulointerstitial fibrosis (92). The vagus nerve-mediated cholinergic anti-inflammatory pathway, as discussed in Section 3.1, represents a neuroimmunological bridge that modulates systemic inflammation but becomes impaired as MASLD progresses.

Mitochondrial dysfunction and oxidative stress are another unifying theme. In hepatocytes, lipid overload induces mitochondrial β-oxidation excess, generating reactive oxygen species (ROS) that promote lipid peroxidation, protein misfolding, and DNA damage. Beyond the liver, ROS and lipid peroxides (e.g., malondialdehyde) circulate systemically, contributing to neuronal oxidative damage, intestinal epithelial apoptosis, and glomerular sclerosis (91, 93, 94). Moreover, oxidative stress activates redox-sensitive transcription factors (e.g., NF-κB, Nrf2) and inflammasomes (e.g., NLRP3), further amplifying inflammatory responses across organs.

Immune system dysregulation, particularly innate immune activation, plays a pivotal role (95). Kupffer cell activation in the liver and macrophage infiltration in adipose tissue are well-established drivers of local inflammation. However, systemic immune cell trafficking-mediated by chemokines (e.g., CCL2, CXCL10) and adhesion molecules-facilitates crosstalk between organs. For instance, liver-derived cytokines promote microglial activation in the brain, while gut-derived endotoxins (e.g., LPS) prime Toll-like receptor 4 (TLR4) signaling in both hepatic and renal tissues (92, 96). Additionally, adaptive immune responses, including Th17 cell differentiation and autoantibody production, have been implicated in the progression of both MASH and extrahepatic autoimmune-like manifestations (95, 96).

Metabolic reprogramming and endocrine dysfunction further integrate systemic pathology. IR is a primary metabolic defect that exacerbates hepatic *de novo* lipogenesis, dysregulates hypothalamic appetite control, impairs gut barrier function, and promotes renal sodium retention and hypertension (90). Adipokine imbalance (e.g., decreased adiponectin, increased leptin) and bile acid signaling alterations also contribute to multisystem crosstalk. For example, bile acids influence cerebral function via FXR receptor activation in the brain, modulate gut microbiota composition, and affect renal electrolyte homeostasis (93).

In summary, these common mechanisms do not operate in isolation but engage in extensive crosstalk, forming self-amplifying feedback loops that drive disease progression across multiple organs. For instance, liver-derived DAMPs and pro-inflammatory cytokines exacerbate systemic inflammation, which in turn impairs the intestinal barrier integrity, leading to increased gut permeability and endotoxemia. This endotoxemia further primes hepatic and systemic immune responses, aggravating liver inflammation and insulin resistance. Concurrently, oxidative stress and metabolic dysregulation perpetuate this cycle by damaging cellular components and promoting lipotoxicity, which fuels further inflammatory signaling and mitochondrial dysfunction. This creates a vicious cycle of metabolic and inflammatory injury that transcends individual organ axes, underscoring the necessity for therapeutic strategies that simultaneously target multiple nodes within this interconnected network. Future research should prioritize integrative models that capture these interactions to guide the development of multisystem therapies.

## 4 Prospect of therapeutic measures

Extensive research has uncovered a range of pathological mechanisms of MASH. Based on these mechanisms, the treatment of MASH is currently categorized into three types: lifestyle intervention, drug therapy, and surgery. Lifestyle interventions are the primary treatment for patients with MASLD/MASH (97, 98). Building on this foundation, pharmacological strategies targeting specific inter-organ pathways and shared pathological mechanisms represent the next frontier in MASH management. A recently published, large-scale prospective cohort study reaffirms the significant benefits of adhering to a healthy lifestyle in the prevention of MASLD (99). If these patients do not respond to lifestyle changes, bariatric surgery is an option (100).

Regarding drug therapy, several groups of drugs aimed at treating MASH based on relevant mechanisms are in the clinical development stage (101–122). Notably, the promising drug classes under investigation are those that target the core inter-organ axes discussed in this review. For instance, FXR agonists primarily modulate the gut-liver axis by restoring bile acid homeostasis and reducing gut-derived inflammation (107, 123). Recent evidence further demonstrates that the FXR agonist INT-767 directly attenuates aberrant basement membrane production by hepatic stellate cells, providing a precise antifibrotic mechanism (124). FGF21 analogues and GLP-1 receptor agonists (GLP-1RAs) exhibit pleiotropic effects across multiple organs; the former improves systemic metabolic regulation and insulin sensitivity (125), while the latter engages the gut-brain-liver axis to control appetite, glycemia, and directly ameliorate hepatic steatosis and inflammation (126). For patients with MASH who have advanced to severe cirrhosis or liver failure, a liver transplant may be necessary (127). The corresponding treatment methods are briefly outlined in **Table 2**.

**Table 2:** Summary of possible interventions for MASLD/MASH and its further deteriorated forms.

Therapy	Type		Example	Effects
Lifestyle Interventions	Weight loss		Diet intervention (98)	Improves metabolism and induces the regression of fatty degeneration
			Exercise intervention (97)	Enhances metabolism and potentially precipitates the resolution of fibrosis
Possible Pharmacotherapies	Metabolic Factors	PPAR agonist	Pioglitazone (101)	Enhances insulin sensitivity and decreases in blood glucose level
		THR-b agonist	Resmetriom (102)	Activates THR-b in the liver selectively and promotes liver fat decomposition
		FXR agonist	Tropifexor (107)	Reduces lipid synthesis and improves fibrosis and steatosis
		GLP-1 agonist	Liraglutide (109)	Reduces blood glucose levels and ameliorates inflammation.
		SCD-1 inhibitor	Aramchol (110)	Reduces *de novo* lipogenesis and enhances fatty acid oxidation
		cholesterol absorption inhibitor	Ezetimibe (104)	Reduces cholesterol absorption and lowers LDL levels in the blood
		Pan-caspase inhibitor	Emricasan (105)	Reduce cell death in liver injury due to apoptosis
		FASN inhibitor	FT-4101 (106)	Inhibits DNL and improves hepatic steatosis
		FGF19/21 analogue	Pegozafermin (108)	Influences sugar intake to regulate energy homeostasis and improve fibrosis
	Immune and inflammatory Factors	TNF-a inhibitor	Pentoxifylline (111)	A nonspecific PDE inhibitor upregulates cAMP
		TGF-b1 inhibitor	Vactosertib (112)	Inhibits TGF-b1 activation
		CCR2/5 antagonist	Cenicriviroc (128)	Inhibits chemokines and disrupts the recruitment of immune cells.
		A3AR agonist	Namodenoson (103)	Protects from liver damage and reduces fibrosis
		BRD4 inhibitor	JQ1 (113)	Blocks HSC activation
		Galectin-3 antagonist	GR-MD-02 (114)	Reverses liver fibrosis/cirrhosis and reduces portal hypertension
		NETs-degrading enzyme	DNase1 (115)	Decomposes NETs both *in vivo* and *in vitro* effectively
		NLRP3 Inflammasome inhibitor	Poria cocos polysaccharides (116)	Inhibits the inflammatory cascade
		TLR4 inhibitor	TAK-242 (117)	Reduces liver inflammation and increases mitochondrial function
		Antioxidant	Vitamin E (101)	Reduces oxidative stress to protect liver cells
	Genetic and epigenetic Factors	ASK1 inhibitor	Selonsertib (122)	Reduces the gene expression related to fibrosis and inhibits excessive apoptosis
		DNA methyltransferase inhibitor	Decitabine (121)	Suppresses the DNA methylation of the PPARγ1 and promotes macrophage activation
		CircRNA Interference	Nanoparticle (120)	Inhibits the progression of fibrosis
		STING inhibitor	Astin C (119)	Alleviates inflammatory response
		Liver-targeted antisense oligonucleotide	Vupanorsen (118)	Reduces hepatic steatosis
	Extrahepatic interactions	Anti-LPS antibody	Imm124-E (59)	Alleviates target Organ damage and lipid profile
		Probiotic	Prohep (63)	Modulates the distribution of bile acids and ameliorates fatty degeneration
		Angiotensin II receptor blocker	Losartan (86)	Reduces PAI-1 production and improves insulin sensitivity
		Fecal microbiota transplantation (64)		Modulates bile acid metabolism and rectifies dysbiosis of the gut microbiota
Surgical Therapies	Surgery		Bariatric Surgery (127)	Improves liver histology
			Liver Transplantation (127)	

The integration of these targeted therapies underscores a paradigm shift from organ-centric approaches to a systems-level view of MASH treatment. Indeed, it is rare for a single drug to comprehensively cure a disease; instead, the combination of multiple drugs targeting different pathways - such as simultaneously addressing metabolic dysregulation, gut barrier integrity, and systemic inflammation - is a promising avenue for future therapeutic development (15, 128). This includes both traditional combinations of distinct drugs and innovative single molecules with inherent multi-target capabilities. A notable example is pemvidutide, a peptide-based GLP-1/glucagon dual receptor agonist exhibiting balanced 1:1 agonist potency at both receptors. This unique profile enables a single molecule to simultaneously activate GLP-1 receptor-mediated metabolic effects and glucagon receptor-driven hepatic actions, representing an integrated pharmacological approach to multi-system disease modulation (129). This approach is supported by early clinical evidence; for instance, a recent network meta-analysis identified the combination of cilofexor (FXR agonist) and firsocostat (ACC inhibitor) as one of the most effective regimens for fibrosis improvement, providing proof-of-concept for synergistic multi-target strategies (130). Furthermore, such multi-targeting agents may themselves serve as foundational components for future combination regimens with other mechanism drugs (e.g., FXR agonists or PPAR agonists), creating even more comprehensive therapeutic strategies.

Additionally, the effectiveness and safety profiles of drugs can differ significantly among individuals, thus necessitating a thorough evaluation and continuous monitoring prior to initiating any treatment. Leveraging experiences from hepatocellular carcinoma treatment, addressing the complexity and heterogeneity of MASLD/MASH will require innovative clinical trial designs and new drug development strategies (131). Future research on combination therapies targeting multiple organ axes should focus on patient stratification and precision medicine.

## 5 Limitations of the current research and future prospects

At present, some progress has been made in the study of the pathological mechanism of the transformation of MASH into liver fibrosis, and the diagnosis and treatment are also discussed from the aspects of metabolism, inflammatory response, and HSC activation. However, at present, there are still some unknown points in the interpretation of this field.

First of all, the specific molecular mechanism underlying the transformation of MASH into liver fibrosis remains unclear, necessitating further studies to elucidate the key molecules and signaling pathways involved in this process. Future research should focus on the comprehensive analysis of multi-omics data to gain a thorough understanding of the pathological mechanisms of MASH, enabling early detection and assessment of the disease and thereby preventing or delaying its progression to liver fibrosis.

Secondly, there are still some limitations in the diagnosis and treatment of MASH. In terms of diagnosis, more sensitive and specific biomarkers need to be developed for early detection and assessment of MASH, in addition to the currently only recognized liver biopsy that reliably differentiates MASH from the aggressiveness of simple steatosis. In recent years, artificial intelligence (AI) has experienced rapid development. Notably, AI applications in liver disease have made significant strides in diagnosis and risk stratification, effectively distilling critical insights from intricate clinical data. Should AI genuinely integrate into clinical decision support systems in the future, it promises not only to conserve substantial human and material resources but also to elevate patient care to new heights (132–135). In terms of treatment, the current treatment of MASH still mainly relies on lifestyle intervention. Surgical treatment such as bariatric surgery is another method for the treatment of obesity in the United States and European countries, but it is not completely effective, and its clinical efficacy and safety need to be further studied to make it suitable for MASLD patients. In terms of drug therapy (100, 109). Due to the complex nature of MASH development and progression, involving oxidative stress, insulin resistance, apoptosis, lipid toxicity, inflammation, and fibrosis, most drug studies targeting various pathways are currently in the clinical trial stage. Looking to the future, given the multifaceted nature of MASH pathogenesis and the often suboptimal response to monotherapy, it may be necessary to explore combination medications and more refined individualized treatment regimens. Future research could adopt successful approaches from hepatocellular carcinoma studies by integrating proteomics, metabolomics, and other multi-omics data with Mendelian randomization methods (136), systematically identifying key molecules driving MASLD multi-organ complications and validating their causal relationships, thus providing a stronger target basis for developing new therapies.

In addition, the community should increase investment in the basic research of MASH, including the improvement of animal models and research methods, with the development of gene editing technology such as CRISPR-Cas9, researchers will be able to create more detailed disease models to simulate the development of MASH (137). This will provide a powerful tool for research to better simulate the condition of MASH in humans, so as to better study its pathological mechanisms and treatment strategies. At the same time, with the increase in the number of MASH patients worldwide, international cooperation will play a more important role in research design, resource sharing and promotion of treatment strategies, and international cooperation and communication should be strengthened to share research results and resources to promote the development of MASH research.

## 6 Conclusions

As an increasingly prevalent hepatic disorder, MASLD/MASH, have garnered escalating attention from medical professionals worldwide due to the intricate interplay of metabolic, genetic, immunological, and extra-hepatic factors underpinning their pathogenesis. Over the past decade, substantial advancements have been made in elucidating the natural history and foundational biology of MASLD/MASH; however, numerous challenges remain unaddressed, and public awareness is lagging. The urgent need for accurate identification, diagnosis, and appropriate management of at-risk and afflicted populations has become paramount. Promising therapeutic approaches and targets are currently under investigation, yet it remains unequivocal that healthy lifestyle modifications and weight management stand at the epicenter of MASLD prevention and control, given obesity’s pivotal role as the primary driver of this widespread liver ailment and its metabolic sequelae. In sum, future research and practice in MASLD must converge on deepened understanding, optimized diagnostic and therapeutic strategies, and intensified lifestyle interventions to effectively combat this global health challenge. Future research should prioritize the integration of multi-omics data and AI to delineate the specific molecular mediators of organ crosstalk, and should champion clinical trials testing combination therapies that simultaneously target multiple extrahepatic axes.
